# Job Insecurity: Differential Effects of Subjective and Objective Measures on Life Satisfaction Trajectories of Workers Aged 27–30 in Germany

**DOI:** 10.1007/s11205-017-1635-z

**Published:** 2017-05-18

**Authors:** Laura Helbling, Shireen Kanji

**Affiliations:** 1 Institut für Bildungsevaluation, Universität Zürich, Zurich, Switzerland; 2 University of Birmingham, Birmingham, UK

**Keywords:** Fixed-term employment, Job insecurity, Subjective indicators, Life satisfaction

## Abstract

Job insecurity has become increasingly evident in European countries in recent years. In Germany, legislation has increased insecurity through erosion of the standard employment relationship. Fixed-term contracts are central to definitions of insecurity based on atypical or precarious work but there is still limited understanding of what creates insecurity and how it affects workers. Drawing on Bourdieu’s thesis that “insecurity is everywhere”, the relationships between subjective and objective measures of insecurity are examined for their impact on the 5-year trajectories of life satisfaction of men and women in the age group 27–30. Latent growth curve analysis of data from the German Socio-Economic Panel for 2010–2014 highlights the adverse and lasting effects of subjective concerns about job insecurity on life satisfaction trajectories. This association cuts across educational groups, with far reaching implications as subjective concerns about job security permeate young worker’s lives well beyond the objective condition of being employed on a fixed-term contract.

## Introduction

Job insecurity has become a key concern in recent decades in Europe. In relation to Germany, the Employment Promotion Act of 1985 (OECD 2004) and the various Hartz Laws signalled a sea change in employment relations and a move away from the standard employment relationship (SER) (Bosch 2004; Brehmer and Seifert 2007). The new types of work that these policies heralded have been collectively and interchangeably termed atypical or non-standard work. They include part-time (particularly very short hours), agency and fixed-term employment. These types of work differ from each other but what unifies them is that they are usually considered to be less secure than the SER and inferior in quality (Keller and Seifert 2013), as indicated by their association with low income (Brehmer and Seifert 2007; Kalina and Weinkopf 2008).

Researchers have argued that the inequalities in job security signified by the increase in fixed-term contracts have become the European counterpart of the USA’s wage inequality (DiPrete 2005). The focus on fixed-term contracts is clear. For example the International Labor Organisation (ILO) placed fixed-term contracts as one of the pillars in its definition of precarious work, a term encompassing a plurality of less secure employment forms (ILO 2012; Keller and Seifert 2013). The concern with insecurity is understandable because of its well-established adverse effects on health. A range of conceptualisations of insecurity, namely employment insecurity (Virtanen et al. 2002), flexible employment (Benach et al. 2000) and precarious work (Quinlan et al. 2001) have all been found to take their toll on health.

The reported effects of job insecurity on work and life satisfaction are less conclusive. Life satisfaction is a cognitive aspect of subjective well-being (Diener et al. 2013) which has been linked to a number of important outcomes, including in the domains of health (as summarized in Erdogan et al. 2012) and job performance (Jones 2006). Evidence suggests that objective and subjective concepts of insecurity diverge in their effects on life satisfaction. De Witte and Näswall’s (2003) study found that temporary work, an objective measure, in itself was not related to lower job satisfaction, but that individuals’ subjective perception of job insecurity affected job satisfaction negatively. Some research has even shown that workers on temporary contracts report higher well-being than that reported by permanent workers, which the authors interpreted as indicative of the worsened conditions of those on permanent contracts (Guest et al. 2010). Indeed, researchers have argued that in contemporary contexts the conditions of those on permanent contracts may not be any more secure than those on temporary contracts (Pedulla 2013; Cappelli and Neumark 2004). Subjective measures provide a view into well-being, which is important of its own right. They are of particular additional value where objective measures provide ambiguous results.

In this article we draw on Bourdieu’s (1998) work on insecurity. Bourdieu specifically identified fixed-term contracts, amongst other insecure work forms, as hindering planning for the future, thus putting life on hold. It is perhaps surprising that Bourdieu pinpointed fixed-term contracts, an objective indicator of insecurity, because he was a key exponent of the argument that subjectivities are key to understanding outcomes and that, as he stated in the title of his article, “Job Insecurity is Everywhere” (Bourdieu 1998). Empirical research certainly bears out the importance of subjectivities in understanding how job insecurity affects life satisfaction (see for example Green 2009; Carr and Chung 2014). However, in the main research on employment insecurity has focused on the detrimental effects of fixed-term contracts which arguably have masked the nature and full impact of insecurity as it is currently experienced. Indeed, our research responds to Baron’s (2013) call to develop an understanding of how changing employment systems impact individual well-being. In this research we probe the effects of both employees’ subjective assessments of their employment security, fixed-term contracts and the interrelationships between these two constructs in their impact on trajectories of life satisfaction. Life satisfaction provides a measure founded on satisfaction with a number of domains in life and thus provides a better guide than other subjective measures of whether life is ‘put on hold’. Moreover life satisfaction provides a measure of the extent to which work conditions impact on workers’ broader satisfaction.

The contribution of the study is twofold. First, we look at workers’ own evaluations of their job security, which serve as one aspect of the insecurity of the macro-system. We investigate to what extent worries about job security permeate the lives of workers in the age range 27–30 and relate to their engagement in fixed-term employment. We focus on this age group because this is the stage in the life course by when most young people in Germany have made the transition from education to employment having followed differing educational and labor market pathways (OECD 2012: 60–63), thus enabling us to compare those with lower and higher educational attainment. Moreover, younger workers in Germany are substantially more at risk of being on fixed-term contracts than older workers (OECD 2016). Second, we examine life satisfaction trajectories which provide an insight into how insecure conditions measured at one point in time impact longer term outcomes. In this task we build on studies which have found unemployment experienced at one point in time and frequent job turnover to have scarring effects, for example in relation to future employment and wages (Bills 1990; Arulampalam et al. 2000; Arulampalam 2000; Niedergesäss 2012). The approach is by intention different from studies which examine how many workers manage to make a transition from fixed-term to permanent employment, which implies permanent jobs to be the yardstick of success. For workers, transitions out of fixed-term work into permanent jobs may not necessarily be satisfaction-enhancing if they were only taken to prevent looming unemployment and repeat fixed-term work (Giesecke and Groß 2003; Gash 2008) but are not the jobs and careers for which these workers had wished and hoped. Moreover, even permanent jobs may not last long. Job insecurity experienced at one point in time may be a marker of an inferior career trajectory which impacts life satisfaction over the longer term. The contribution of this study is an attempt to gauge if felt job insecurity and objective job insecurity, which is measured as fixed-term employment, puts life on hold (Bourdieu 1998) in the sense of leading to a relatively lower trajectory of life satisfaction.

## Theoretical Perspectives

Bourdieu (1998) used the term precarity (*precarité)* to explain the existence of a pervasive and contemporary social threat that is all-present in high-income economies. He went so far as to describe it as “establishing of a generalized and permanent state of insecurity that leads towards obligating workers into submission and the acceptance of exploitation” (1998: 82). If this state of insecurity is ubiquitous, then the precariously employed, those in contingent or atypical employment, may not be in a worse position than those whose work is governed by the SER. The conditions of the SER may have deteriorated, at least in terms of the security it affords, to match the conditions of work that is objectively precarious (Pedulla 2013). As Marchart (2013) sees it, *precarization processes* extend the reach of precarity to affect those employed on secure terms. The implication is a convergence in how workers in each of these objective conditions subjectively view their conditions; for example, Cappelli and Neumark (2004) demonstrated the equivalent likelihood of involuntary labor-market turnover in the core and periphery of the dual labor market, undermining the thesis of the segmented labor market. Essentially, the argument is that an insecure macro-environment induces worries about job security across all workers. This argument leads to the expectation of a lack of difference in the effects on well-being trajectories of being on a fixed-term or permanent contract, an expectation which is not suitable for deriving hypotheses. We therefore formulate our expectations in the form of a research question.

### Research Question 1

Are employees in insecure work, as indicated by fixed-term contracts, any more worried about job insecurity than those on permanent contracts?

In explaining how the current system of insecurity sustains itself, both Bourdieu (2000) and Burawoy (2012) have argued that the objective conditions of labor exploitation diverge from workers’ subjective evaluations. As Burawoy explained, the non-monetary rewards workers derive from the very fact of having a job lead them to comply with their objective exploitation. Workers do not fully take into account the inferiority of their objective conditions. The subjective and the objective are closely related, in that they support a system of exploitation but they are likely to have different effects on workers’ self-perceptions. Thus, contingent work, which is generally inferior in its wages and prospects (Kalleberg 2011), might not have the expected negative effect on self-reported life satisfaction.

Applying this idea further, it is possible to see how young people with expectations of a future career that is superior to their current objective condition of a fixed-term contract might not experience adverse effects on their well-being. The objectively inferior conditions of fixed-term contracts in Germany have been established in previous research: they are associated with lower occupational status and wages (see Gash and McGinnity 2007; Giesecke 2009; Giesecke and Groß 2004; Mertens and McGinnity 2003), higher risk of subsequent unemployment (Giesecke and Groß 2003) and a higher risk of continuing in serial temporary jobs (see Gash 2008; Giesecke and Groß 2003). Although young workers are at a higher risk of insecure work (Blossfeld et al. 2008), they may accept that uncertainty is an aspect of a necessary stage of their career trajectory. Workers who perceive that precarious work can lead to something better may feel empowered to be in such work or, at least, not feel badly about it. As in Bourdieu’s (2000) and Burawoy’s (2012) explanations, workers derive benefits from work that are additive to its current value. Therefore, the effects of insecure contractual status on well-being may diverge from the effects of subjective experiences and fears.

The evidence of whether precarious work in Germany acts as a conduit to better work is mixed (Gash 2008; Hohendanner 2010; Scherer 2004). Men working on fixed-term contracts, for example, suffer a wage penalty and are somewhat more likely to become unemployed or to be re-employed on a fixed-term contract but these differences dissolve over time (Gash and McGinnity 2007). The higher subsequent unemployment risk of young workers who start work on a fixed-term contract diminishes over time (McGinnity et al. 2005), and no negative effects of the initial fixed-term contract are found on later occupational positions or prestige (McGinnity et al. 2005; Scherer 2004) while initial wage differentials diminish (Gebel 2010). Many workers in low-paid temporary jobs in western Germany experience high wage growth (Mertens and McGinnity 2003). The evidence seems to show that there is a high probability that fixed-term contracts act as stepping-stones, a situation which co-exists with the high probability of fixed-term contracts leading to unemployment (Gundert and Hohendanner 2014). Little is known about whether fixed-term contracts impact workers’ life satisfaction in Germany and how these effects evolve and endure over time, an important shortfall in our understanding that this article aims to address.

The evidence suggests that contractual insecurity does not necessarily impede young workers’ longer-term occupational integration and career advancement but it is possible that there is a strong educational gradient to the future prospects of those on fixed-term contracts. In many European countries, low-skilled workers face a higher relative risk of holding a fixed-term contract than higher-skilled people. Germany differs in that those with high educational attainment have been found to have the same or even a higher risk of being employed on a fixed-term contract (Gebel and Giesecke 2011; Mertens and McGinnity 2003), especially in relation to new labor-market entrants (McGinnity et al. 2005). There could be a dichotomy in the effects of fixed-term contracts for those with lower and higher educational attainment, consonant with Kunda et al.’s (2002) thesis of a dichotomy between low-skilled and highly skilled workers in contingent employment (see also Marler et al. 2002). This dichotomy could be reinforced by diverging years of experience in the labor market by educational qualification which would imply that someone who was 29 with low educational attainment would have experienced more years in the labor market than someone with higher qualifications and therefore interpret the experience of insecurity differently from one of their peers with higher education.

The psychological contract provides further insight into how objectively inferior conditions could have little impact on well-being (De Witte 2005). Again, expectations play a critical role, but they operate differently, in that objective conditions suppress what workers might expect. In a sense, this is the problem of adaptive preferences, for example, when preferences reflect feasibility and constraint (Sen 1999) or discrimination (Nussbaum 2000). Empirical research seems to bear out that those on temporary contracts have lower expectations, which mediate their job insecurity experiences such that they do not experience negative well-being effects (De Witte 2005; Khattab and Fenton 2009). The psychological contract between workers and employers differs for temporary as opposed to permanent workers, who expect more from their employers, as research studies using data for Belgium (De Cuyper and De Witte 2006) and seven European countries (Guest et al. 2010) has suggested. Thus as expectations between permanent and temporary workers may differ, those on fixed-term contracts may not experience lower life satisfaction than those in permanent work.

Although these theories about the connections between subjective and objective conditions differ in their proposed mechanisms, they lead to the expectation that fixed-term contracts are not necessarily related to a lower unfolding of life satisfaction over time. Indeed, some workers may be more satisfied with their work and lives than their objective conditions would imply, because they factor in a better work future. Concern about insecurity may cut across fixed-term and permanent contracts following Bourdieu’s (1998) theorisation that job insecurity (or precarité) is everywhere and is undermining workers’ welfare. This leads us to ask:

### Research Question 2

Do subjective worries undermine life satisfaction over time regardless of whether workers are employed on objectively insecure terms?

In addition to the potentially negative effect of worries, it is possible that those who do not envisage their fixed-term work acting as a bridge to better employment experience worse well-being outcomes, which are exacerbated by being on a fixed-term contract. A subsidiary question that probes further into the relationship between fixed-term contracts and subjective insecurity relates to their combined effects.

### Research Question 2a

Is the detrimental and enduring effect of workers’ worries on life satisfaction heightened by the objective experience of contractually insecure work?

## Data

The German Socio-Economic Panel (GSOEP) is a longitudinal survey of households and individuals conducted annually since 1984. It consists of the core sample and various refreshment and supplementary samples covering, for example, migration, family and wealth. This analysis makes use of all these samples and applies the sample weight supplied by the GSOEP to adjust for over- and under-sampling.

We use data from 2010 to 2014 to focus on employees born between 1980 and 1983, whose ages ranged from 27 to 30 in the first year of observation. Examining this age group allows us to study the phase of labor market experiences that encompasses those with low and high educational qualifications. The unweighted number of observations in this age range is 1874 young people, of whom 739 are men and 1135 are women. Migrants account for 506 people in the sample. We excluded from the sample those who were not working in regular employment, such as those who were in education or training, unemployed, in government subsidized jobs, the economically inactive, and people with special needs. Among the excluded observations there were 291 women on maternity leave and another 84 women with children who reported they were not working in 2010. The reason for the relatively high number of women on maternity leave is that data from a family-focused survey were incorporated into the SOEP data covering the period from 2010. We also excluded 51 young adults who were self-employed and 48 people who reported that they did not have a labor contract, plus 40 young people who were working no more than 20 h and also still in education or training. The final analysis sample consisted of 980 young employees, of whom 461 were women and 519 were men; 751 of these employees held German nationality and were born in Germany with no parental migrant history, while 229 had what is referred to in Germany as “a migration background”. Based on the ISCED 2011 classification, only 86 young workers had attained only a very low level of qualifications (ISCED 1–2), 654 workers had a medium education (ISCED 3–5), while 234 were highly educated (ISCED 6–8) (6 missing values). Overall, in this age category of 27–30, 214 workers were employed on a fixed-term contract in 2010 while 762 were employed on a permanent contract (4 missing values).

Employment on fixed-term contracts (19% of women and 18% of men in the age category) affects men and women about equally, as was found in an earlier period by Brinkmann et al. (2006: 26), who reported that 13.1% of female workers and 12.5% of male workers were on fixed-term contracts across all age groups. There are differences in the exposure of migrants and non-migrants to employment on fixed-term contracts, with non-migrants overrepresented in fixed-term work (20 vs. 13%). Probing this difference further, we found that a comparison between those born in Germany and those born elsewhere narrowed the gap so that those born in Germany were four percentage points more likely to be on a fixed-term contract. Regional disparity is evident. A higher proportion of young people in eastern Germany (26%) are on a fixed-term contract than in western Germany (17%). Educational attainment differences are also notable but not as one might expect: 27% of those with high educational attainment (ISCED 6-8), while 18% of those with low educational attainment (ISCED 1–2) are employed in fixed-term work and 13% of young workers who have medium educational qualifications (ISCED 3–5) hold a fixed-term contract (as the group of young workers holding only low educational qualifications is small, caution is needed regarding the estimate of fixed-term employment for this group)

## Variables

Contractual status is coded as 1 if the individual is employed on a fixed-term contract, and 0 if he or she is employed on a permanent contract. The subjective measure of job insecurity uses the answer to the question, “*Are you concerned about your job security*?” This item is a measure of affective job security (see Chung and Mau 2014 for a full discussion of the categorization of types of job insecurity). To draw a clear distinction between those who are concerned from those who are not, we coded the variables as 1 if the person is very or somewhat concerned, and 0 if he or she is not concerned. Recognizing the potential overlap between objective and subjective measures, we also include an interaction term between being on a fixed-term contract and being concerned about job security. All measures of employment insecurity relate to the initial year of observation in 2010. The variable for life satisfaction is based on the question, “*How satisfied are you with your life, all things considered?”* Responses are coded from totally unhappy (coded 0) to totally happy (coded 10). The estimated life satisfaction trajectories use data from five consecutive years. Previous studies have found considerable stability in this single item measure over shorter rather than longer time intervals (Fujita and Diener 2005). Gender is a dichotomous variable coded 0 for female and 1 for male. Living with a partner is coded 0 if the respondent is not living with a partner and 1 if the partner lives in the same household. Having a child under 18 living in the household is coded 1 if there is a child under 18 living in the household and 0 otherwise. Employment status indicates whether the person works full-time, regular part-time, or marginal/irregular part-time, which are self-reported categories. Furthermore, we include a binary variable capturing previous unemployment exposure in the respondent’s career as previous experiences of unemployment are known to be associated with life satisfaction (Lucas et al. 2004). A variable is included for region distinguishing eastern and western Germany as it is known that life satisfaction is lower in eastern Germany (Easterlin and Plagnol 2008). Work experience is measured in separate variables as years and months of full-time and part-time work experience while tenure in the current firm forms an additional control. Education is classified as low, medium, and high levels of education using the International Standard Classification of Education (ISCED 2011). We code those whose highest level of attainment is ISCED level 2 and below as having a low educational achievement, those who have attained ISCED level 3–5 (including upper-secondary to short cycle tertiary education) as having a medium level of attainment, and those who have attained ISCED level 6–8 (including bachelor and master degrees as well as doctoral levels) as having a high level. We further include a dummy variable coded 1 if the person has a direct or indirect migrant background and 0 otherwise.^1^ In addition, we include hourly wage rates^2^ as a measure of job quality and because income has been associated with life satisfaction (Diener and Oishi 2000).

## Analytical Strategy

To investigate the first research question about the overlap and connections between objective conditions and subjective perceptions of job insecurity, we report cross-tabulations produced using Stata 14 in Table 1. The reported design-based F statistic, which accounts for the survey design, indicates whether there is a statistically significant relationship between objective and subjective measures of job insecurity.

**Table 1:** Worries about job loss and contractual insecurity

	Permanent contract	Fixed-term contract	Total
Not worried	53% (344)	35% (85)	49% (429)
Worried	47% (414)	65% (125)	51% (539)
Total	100% (758)	100% (210)	100% (968)

Percentages are weighted values, number in brackets refers to unweighted sample sizes

Design-based F (1, 653) = 3.9, significant at *p* ≤ 0.05

We next explore Research Question 2 and 2a which relate to whether contractual insecurity, again defined in subjective and objective terms, manifests in differential well-being trajectories. Latent growth curve modelling is used to distinguish individual well-being trajectories in relation to job insecurity over a period of five years. Growth curve analysis models allow us to examine the variation between people in the trajectories of well-being over time. Multi-level and Structural Equation Models (SEM) can produce equivalent results, but we chose the SEM approach because it is better suited to the estimation of latent variables that estimate and remove the effects of measurement error in the predictors or the outcomes (Curran et al. 2010).

**Fig. 1:**
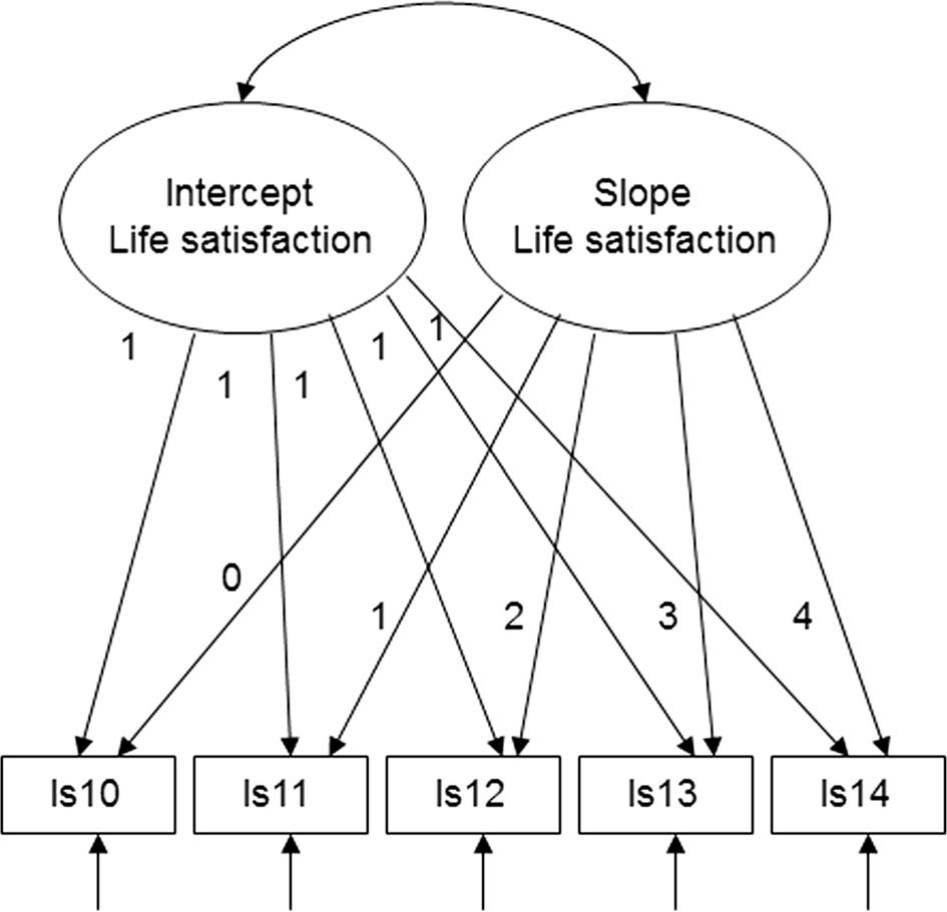
Unconditional growth curve model. ls10-14 stand for life satisfaction in each of the years 2010–2014

The process of running the latent growth curve model involves two steps. First, we estimate an unconditional growth curve model without explanatory variables to assess the average trajectory of life satisfaction of young workers in Germany between 2010 and 2014. The latent intercept represents average initial life satisfaction in 2010, and the latent slope parameter represents average rates of change in life satisfaction over the 2010–2014 period. The variances of the latent growth parameters indicate whether there is inter-individual variation in initial life satisfaction and in the rate of change of life satisfaction across young workers (see Bollen and Curran 2006; Byrne 2012). The unconditional SEM model is shown in Fig. 1 and presented below.

Unconditional model:

$$\begin{aligned} & {\text{Trajectory}}\;{\text{equation:}}\,Y_{it} = \alpha_{i} + \lambda_{t} \beta_{i} + \varepsilon_{it} \\ & {\text{Unconditional}}\;{\text{intercept}}\;{\text{equation:}}\,\alpha_{i} = \alpha_{0} + \zeta_{{\alpha {\text{i}}}} \\ & {\text{Unconditional}}\;{\text{slope}}\;{\text{equation:}}\,\beta_{i} = \beta_{0} + \zeta_{{\beta {\text{i}}}} \\ \end{aligned}$$

The vector *Y*_*it*_ includes the repeated observed measures, that is life satisfaction scores for individual i over time t. Focusing on life satisfaction over the time period of 2010-2014, the individual (latent) intercept α_*i*_ represents the initial life satisfaction score for individual i in the first year of observation, which is in 2010, while the individual (latent) slope parameter β_*i*_ represents the individual rate of change in life satisfaction over the period observed, which is from 2010 to 2014. The vector λ_*t*_ includes pre-defined regression weights allowing the estimation of the (latent) intercept and slope parameters. Assuming a linear trend based on 5 time points (2010–2014) with equally spaced intervals, λ_*t*_ includes the values (0, 1, 2, 3, 4). Estimating mean trajectories, α_0_ represents the mean life satisfaction of the individuals investigated in 2010, while β_0_ represents the mean rate of change from 2010 to 2014. The disturbances $\zeta_{{\alpha {\text{i}}}}$ and $\zeta_{{\beta {\text{i}}}}$ (with means of zero and variances of $\zeta_{{\alpha {\text{i}}}}$, $\zeta_{{\beta {\text{i}}}}$ as well as their covariance) represent individual deviations from the mean intercept α_0_ and mean slope β_0_. The variances of the latent growth parameters hence indicate whether there is inter-individual variation in initial life satisfaction and in the rate of change of life satisfaction (see Bollen and Curran 2006).

Including predictors of latent growth parameters in the growth curve model (leading to the conditional model), the intention is to explain inter-individual variability in latent intercept and latent slope parameters describing subjective life satisfaction trajectories.

**Fig. 2:**
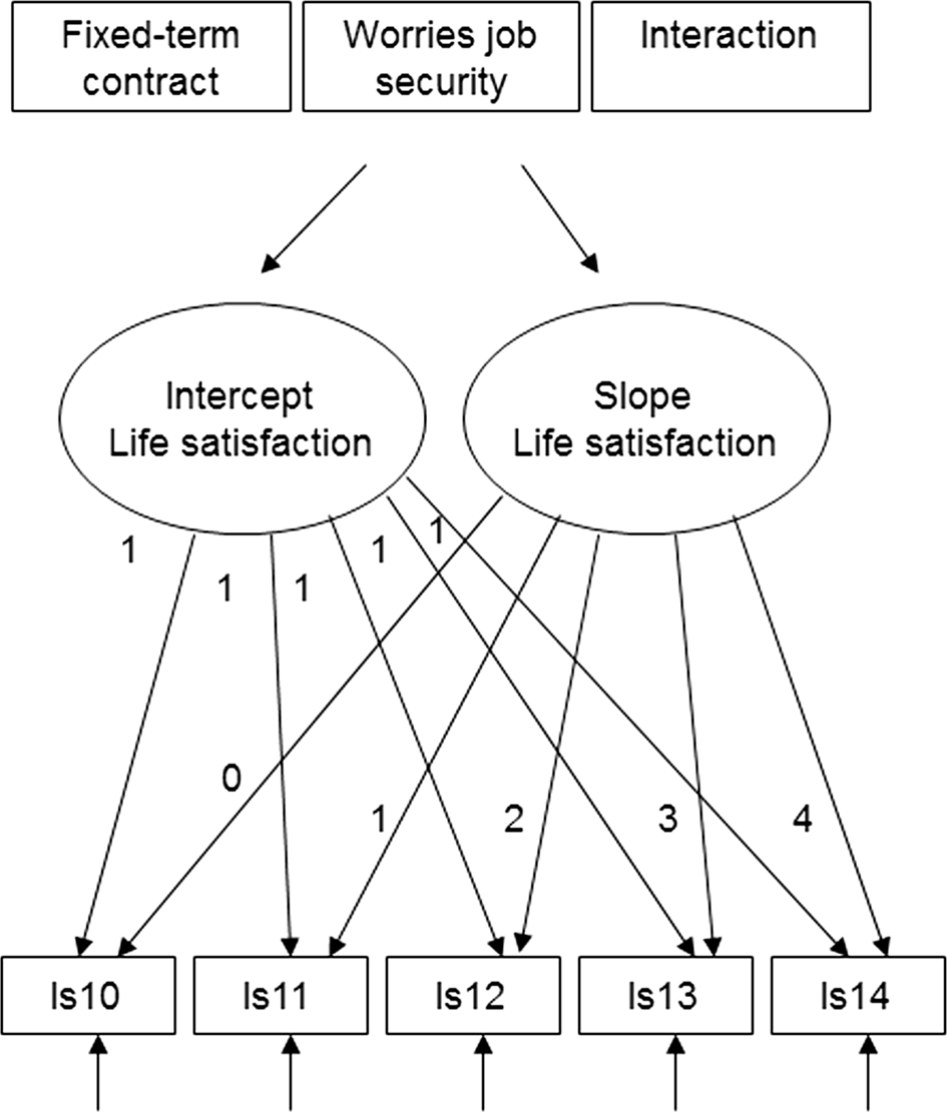
Conditional growth curve model. ls10–14 stand for life satisfaction in each of the years 2010–2014 For illustration purposes, the covariance among growth parameters as well as paths from each explanatory variable and control variables to latent growth parameters are not shown. Underlying this conditional growth-curve model is the baseline model illustrated in Fig. 1

The second step is to estimate a conditional growth curve model introducing fixed-term contracts and concern about job security as predictors of the latent intercept and slope parameters, based on the baseline model established in the first step. This model enables us to test whether these types of insecurity are related to differential life satisfaction trajectories. The conditional model is presented in Fig. 2, while the equations for this model are presented below.

$$\begin{aligned} & {\text{Conditional:}}\,{\text{intercept}}\;{\text{equation}}:\alpha_{i} = a_{\alpha } + b_{\alpha 1} x_{1i} + b_{\alpha 2} x_{2i} + \zeta_{{\alpha {\text{i}}}}^{*} \\ & {\text{Conditional}}\;{\text{slope}}\;{\text{equation:}}\,\beta_{i} = a_{\beta } + b_{\beta 1} x_{1i} + b_{\beta 2} x_{2i} + \zeta_{{\beta {\text{i}}}}^{*} \\ \end{aligned}$$

$x_{1,2}$ represent explanatory variables, such as subjective and objective job insecurity, which predict the latent intercept and slope parameters. The coefficients $b_{\alpha 1,2}$ and $b_{\beta 1,2}$ represent the effects of the explanatory variables on the latent growth parameters. Significance testing regarding the coefficients $b_{\alpha 1,2}$ indicates whether there is an impact of the explanatory variables (subjective and objective job insecurity) on the latent intercept (initial life satisfaction in the year 2010) while significance testing of $b_{\beta 1,2}$ indicates whether these explanatory variables have an impact on the latent slope, that is, the rate of change in life satisfaction. The terms $a_{\alpha ,\beta }$ are regression intercepts while $\zeta_{\alpha ,\beta }^{*}$ represent disturbances. Variances of these disturbances no longer represent variances of the latent slope parameters (as is the case for the unconditional model) but represent individual noise after conditioning the latent growth parameters on explanatory variables. The intercept and slope equation of the conditional model can be thought of as being subsumed into the trajectory equation reported above, leading to a single combined equation (see Bollen and Curran 2006).

The interaction term enables us to gauge if there is an additional effect on life satisfaction from the interaction between objective conditions and subjective feelings of insecurity, an impact that goes beyond their individual effects.

The latent growth-curve modeling is executed using MPlus and is based on full information maximum likelihood (FIML) (Enders and Bandalos 2001) and robust maximum likelihood (MLR). We take into account the complex survey design by using the psu and strata variables, as well as a sampling weight provided by the GSOEP. Model fit is indicated by χ^2^-values. As χ^2^-test statistics strongly depend on sample size (see Bollen and Curran 2006: 44–45), we base our assessment of model fit on further fit indices, such as the comparative fit index (CFI) (Bentler 1990), as well as the root mean square error of approximation (RMSEA) (see Browne and Cudeck 1993).

## Results

### Overlap: Objectivity and Subjectivity Concerning Contractual Insecurity

While 18% of employees in the 27–30 age range hold a fixed-term contract, 51% of the whole group of workers in this age range worry to some degree about their job security (see Table 1). Anxiety about job security permeates the lives of workers in this age category to a much greater extent than the condition of having a fixed-term contract. Our first research question was built on the argument that “precarity is everywhere” and may not necessarily differ between those who are on fixed-term and permanent contracts. Findings reported in Table 1 do not support the notion that precarity is equally spread between those on fixed-term contracts and permanent jobs in that a much higher share of workers in fixed-term contract work are worried about their job security based on the design-based F statistic, a measure of association between the two binary measures of subjective and objective insecurity (significant at *p* ≤ 0.05). However, it is at the same time also striking that such a high proportion of those on permanent contracts also experience worries about job security.

The subsequent transitions between fixed-term and permanent jobs and unemployment of young workers employed in 2010 are diverse and complicated involving transitions between fixed-term and permanent jobs as well as self-employment and unemployment. About 46% of the sample employed on a fixed-term contract in 2010 transitioned to a permanent job at some point over the 5-year period (without intermediate unemployment, but they may have subsequently left this permanent employment), 41% of the sample remained employed on a fixed-term basis as long as we observe them. About 13% of those employed on a fixed-term contract experienced some spell of unemployment or non-employment (not including maternity-leave or further education) during the period observed. Of those employed on a permanent contract in 2010, the majority of about 82% of young workers remained employed on a permanent contract as long as we observed them over the period investigated, about 12% moved from a permanent contract to a fixed-term contract while about 6% experienced some unemployment or non-employment. The employment trajectories of the sample are even more diverse than these figures suggest: they include movements from permanent employment to unemployment and subsequent re-engagement in fixed-term work or from fixed-term work to a permanent position followed again by fixed-term job or fixed-term employees experiencing some unemployment then gaining a foothold in a permanent position, or workers may also become self-employed. The interest of this study does not lie in disentangling all of these diverse objective trajectories. Rather, its focus is in whether fixed-term employment and felt job insecurity “put life on hold” (Bourdieu 1998) with young workers experiencing their current labor market integration and subsequent trajectories as less rewarding as reflected in their satisfaction with their lives.

### Unconditional Growth Curve Model

The unconditional latent curve model matched the data well, according to goodness-of-fit statistics (MLRx^2^ = 18, *df* = 10, χ^2^/*df* = 1.8, CFI = 0.97, RMSEA = 0.029, *P*-close = 0.96) (see Fig. 1).

In Fig. 1, following structural equation convention, ellipses represent unobserved factors (latent growth parameters), rectangles represent observed factors (repeated observed measures for life satisfaction over the 2010–2014 period), single-headed arrows represent regression paths, including the values of the introduced regression weights, and curved double-headed arrows represent covariances. Arrows without sources denote the residuals.

The results point to limited change in mean scores of life satisfaction over the 2010–2014 period for 27–30 year old employees (see Table 2). Masked by this apparent uniformity in experience is the significant inter-individual variability with regard to the latent intercept, which indicates that there are differences between individuals in initial life satisfaction. We find however no significant variation in the latent slope parameter, indicating that change over time is similar across people investigated.

**Table 2:** Mean scores of life satisfaction for workers aged 27–30 in the SOEP 2010–2014

Mean scores	2010	2011	2012	2013	2014	Rate of change (mean slope)
Life satisfaction	7.5	7.5	7.4	7.6	7.4	0.01 n.s.

### Conditional Growth Curve Model

Introducing measures of objective and subjective job insecurity and their interaction as predictors into the baseline growth curve model described above we estimate the conditional latent growth curve model represented in Fig. 2. The aim of this approach is to explore and describe the life satisfaction trajectories associated with subjective and objective notions of job insecurity.

In order to understand the results better we also control in an additional model for factors which are likely to be associated with objective and subjective job insecurity and well-being. Comparison of models with and without controls (see Table 3, M1 and M2) enables us to gauge whether the effects for job insecurity found in the model with no additional controls are driven by or even confined to other factors which could be the drivers of insecurity. Previous research has established educational level, gender, migration, the presence of children, having a co-resident partner and region as key predictors of well-being (see for example Easterlin and Plagnol (2008) and Frijters et al. (2004) in relation to Germany), all factors that could also be related to work insecurity. In relation to job characteristics and different work histories of young employees we add controls for full-time and part-time work experience, and tenure with the current employer as well as employment status (full-time, part-time and irregular/marginal part-time), because part-time and mini-jobs (Keller and Seifert 2013) are characteristics of peripheral jobs that could overlap with fixed-term employment (Giesecke and Groß 2003) and fears about job security. In order to account for the association between non-standard work and inferior-quality jobs (Brehmer and Seifert 2007; Kalina and Weinkopf 2008), we include hourly wage rates, Previous unemployment experience is included because the effects of unemployment on well-being are well documented (Jahoda 1988). Those who were previously unemployed are more often in insecure work at re-entry (Giesecke and Groß 2003, 2004) and potentially suffer heightened fears about future job loss. In Table 3 we report the main findings from the models with (M2) and without (M1) controls. It is notable that we obtain similar results across both models which indicates that the results for fixed-term contracts and subjective employment insecurity are robust to the inclusion of this wide range of additional variables.

**Table 3:** Job insecurity and life satisfaction

	Effect (M1)	SE	Effect (M2)	SE
Intercept life satisfaction	7.62***	0.09	7.09***	0.37
Slope life satisfaction	0.02	0.02	0.19(*)	0.11
*Intercept life satisfaction*				
Fixed-term contract	0.33	0.41	0.35	0.42
Worries about job security	−0.34*	0.16	−0.26(*)	0.16
Interaction	−0.50	0.47	−0.31	0.45
Partner	–	–	0.46**	0.16
Wage	**–**	**–**	0.03*	0.01
Previous unemployment	**–**	**–**	−0.33(*)	0.18
*Slope life satisfaction*				
Fixed-term contract	−0.04	0.07	−0.05	0.07
Worries about job security	−0.01	0.05	−0.02	0.06
Interaction	0.04	0.10	0.00	0.11

Significant effects are highlighted in bold and discussed in the results section

(*) ≤0.1; * ≤0.05; ** ≤0.01; *** ≤0.001

M1 (no controls), N = 966: MLRx^2^ = 28.8, *df* = 19, x^2^/*df* = 1.5, CFI = 0.97, RMSEA = 0.023, *P*-close = 1.00

M2 (controls added), N = 857: MLRx^2^ = 111.7, *df* = 61, x^2^/*df* = 1.8, CFI = 0.90, RMSEA = 0.031, *P*-close = 1.00

Control variables: gender, migrant status, education, children at home, partner, previous unemployment, work experience (full-time and part-time), firm tenure, full-time vs. regular/marginal part-time, wage, region

Only significant effects (*p* ≤ 0.1) of control variables are displayed in M2

Focusing first on the effects of commencing the period on a fixed-term contract the lack of a significant result for the latent intercept of life satisfaction in Table 3 and the positive direction of the point estimate shows that fixed-term contract work is not per se associated with initially lower life satisfaction. The lack of a significant association between fixed-term work and the latent slope for life satisfaction further indicates that those initially employed fixed-term who did not worry about their job security also do not experience a decline in life satisfaction over the period investigated.

Table 3 reports that concern about job security is significantly negatively related to the latent intercept of life satisfaction but not with the latent slope of life satisfaction, meaning that subjective insecurity is related to the initial status but not the process of change in life satisfaction over time. Since the variance of the latent slope parameter was estimated to be not significant, there is little purpose in including further covariates in the rate of change. Those initially worried about job loss in 2010 report lower initial life satisfaction and do not experience any closure of the initial gap in life satisfaction between themselves and those who did not worry. This result show that workers’ feelings about their job security, even if employed on objectively secure terms, undermine their life satisfaction and these effects last over the time span we observe (Table 4).

**Table 4:** Job insecurity and life satisfaction disaggregated by education group

	Medium/low education (N = 728)		High education (N = 233)	
	Effect	SE	Effect	SE
Intercept life satisfaction	7.53***	0.14	8.00***	0.17
Slope life satisfaction	0.01	0.03	0.00	0.04
*Intercept life satisfaction*				
Fixed-term contract	0.83	0.55	−0.31	0.31
Worries about job security	−0.27(*)	0.16	−0.46*	0.21
Interaction	−0.97	0.60	0.13	0.42

Significant effects are highlighted in bold and discussed in the results section

The latent slope variance is constrained to zero for model fit purposes

(*) ≤0.1; * ≤0.05; ** ≤0.01; *** ≤0.001

MLRx^2^ = 93.1, *df* = 48, x^2^/*df* = 1.4, CFI = 0.87, RMSEA = 0.044, *P*-close = 0.75

Contrary to our prior expectations as formulated in the Research Question 2a we find no significant interaction effect between objective and subjective notions of job insecurity in their effect on well-being. On average, the negative effect on life satisfaction that fear of job loss brings is not significantly heightened by holding a fixed-term contract, even though these workers (employed on fixed-term contracts and worried about job security) have on average the lowest life satisfaction.

Focusing in addition on significant effects (*p* ≤ 0.05) of control variables included in M2 we find young workers who have a partner to be more satisfied with their lives than those who are single. The formerly unemployed have lower initial life satisfaction. Wage is positively related to life satisfaction. We also find those in the low and medium educated categories to have lower life satisfaction than the more highly educated, but differences are not significant.

In order to understand better the above results we further explored the possibility of educational differences through a separate multigroup analysis of two educational groups: the low and medium educated (ISCED 1–5) and highly educated (ISCED 6–8). Worries about job insecurity, which are not confined to permanent jobs, go together with lower life satisfaction across both educational groups (effect is significant at *p*≤ 0.05 for highly educated group while it is only significant at *p* ≤  0.1 for the low/medium educated group). All in all our results suggest that worries about job security also exist among highly educated and also relate to lower initial life satisfaction which endures over a 5-year period.

## Discussion and Conclusion

In this study we examined how subjective and objective concepts of job insecurity affected the 5-year trajectory of young workers’ life satisfaction in Germany. The results show that subjective insecurity is clearly related to a diminished starting point for life satisfaction, which is not bridged over the 5-year period we examine. Therefore the adverse starting point for these workers endures, potentially relating to a scarring effect of subjective insecurity as has previously been found for unemployment. The association between subjective insecurity and well-being is concerning because subjective insecurity permeated young people’s lives (51% of workers were concerned about job security) to a great extent. The widespread existence of subjective fears about job security and their negative well-being effects lend support to Bourdieu’s (1998) argument that worries permeate the environment to the detriment of workers. The results do not seem to indicate that fixed-term contracts are a strong indicator of demarcation between the core and periphery (Doeringer and Piore 1971) in that concern about job security is experienced by a high proportion of those on permanent contracts, workers who should be in the core.

Previous research conducted in the United States has also found that insecure conditions extend to those on ostensibly secure contracts (Pedulla 2013; Broschak and Davis-Blake 2006). Furthermore, the results suggest that concern about job security adversely affects both those with higher and lower educational attainment. This result in relation to education is all the more striking because previous studies have found education to be a good predictor of subjective insecurity with those with higher levels of education being more protected from adverse effects (Green 2009). Subjectivities have often been neglected in studies of non-standard work or have been taken as inherently reflected in objective conditions, which our study suggests is not the case. Indeed we find grounds that a kind of “subjective precarisation” has taken place. Subjectivities are not and should not be the only yardstick for evaluating insecure work conditions, but we join Chung and Mau (2014) and Lübke and Erlinghagen (2014) in arguing they can help to deepen our understanding of how changing employment systems affect workers’ lives. Moreover using life satisfaction trajectories as an indicator of well-being adds to the understanding gained from more objective measures such as wages (Diener et al. 2013).

Clearly subjectivities are important in understanding how well-being unfolds over time. At the same time the evidence shows that having a fixed-term contract does not negatively affect well-being in the absence of subjective worries, which applies to 35% of those on fixed-term contracts (see Table 1). This group of young workers on fixed-term contracts do not worry about their job security and do not suffer diminished life-satisfaction. Potentially these are the workers who expect fixed-term contracts to act as stepping stones or who have already factored in diminished expectations about their working prospects.

We employed latent growth curve modeling to examine trajectories of life satisfaction rather than looking at snapshots in time. Our interest was to gauge differences in initial life satisfaction and evolution of life satisfaction by objective and subjective insecurity at the stage when most workers had made the transition to the labor market. Gender differences were not the focus of our research, in part because fixed-term contracts are not obviously gendered in the same way as part-time work and mini-jobs. Yet, we recognize that the wider phenomena of atypical and low-wage work in Germany are highly gendered and that this is an area in need of much further work.

Insecurity is a feature of work for the youngest workers in Germany, but we addressed the next stage in the labor market, looking at how insecurity affects the well-being trajectories of those in the 27–30 age group. The challenge of future research will be to continue this focus on the effects of insecurity over the life-course, particularly for younger cohorts that have been highly exposed to contract insecurity in the first stage of the transition from education to employment. Furthermore, it is necessary to recognize the differentiated and changing meanings of contract status: that for some highly educated workers a fixed-term contract is not detrimental per se, while for those with lesser educational attainment, a fixed-term contract negatively impacts well-being. What is really notable is that the subjective feeling of job insecurity negatively impacts workers as an overall group, an effect that seems to be present separately for highly-educated workers and those with low and medium education. Moreover, the lower initial life satisfaction of those who experience subjective job insecurity at one point in time is lasting.

## Footnotes

1 The GSOEP defines migrants as having a direct or indirect migration background, the latter referring to a person born in Germany whose parents were migrants.

2 Hourly wage rates are derived from monthly gross salary and actual weekly working hours, including overtime. Monthly Gross salaries are divided by monthly hours to obtain hourly wage rates, following Kalina and Weinkopf (2008: 467) although excluding retrospective data on special payments. We use the generated variable Labgro to compute hourly wage rates, 6.5% of the monthly incomes are imputed. Excluding the imputed income values does not change the results, and therefore we keep cases with imputed income values.
